# Management of secondary chronic headache in the general population: the Akershus study of chronic headache

**DOI:** 10.1186/1129-2377-14-5

**Published:** 2013-01-30

**Authors:** Espen Saxhaug Kristoffersen, Christofer Lundqvist, Kjersti Aaseth, Ragnhild Berling Grande, Michael Bjørn Russell

**Affiliations:** 1 Head and Neck Research Group, Research Centre, Akershus University Hospital, PO Box 95,, 1478, Lørenskog, Norway; 2 Department of General Practice, Institute of Health and Society, University of Oslo, Oslo, Norway; 3 Institute of Clinical Medicine, Campus Akershus University Hospital, University of Oslo, Nordbyhagen, Norway; 4 HØKH, Research Centre, Akershus University Hospital, Lørenskog, Norway; 5 Department of Neurology, Akershus University Hospital, Lørenskog, Norway

**Keywords:** Secondary chronic headache, Chronic migraine, Medication-overuse headache, Health care utilisation, General population

## Abstract

**Background:**

The prevalence of secondary chronic headache in our population is 0.5%. Data is sparse on these types of headache and information about utilisation of health care and medication is missing. Our aim was to evaluate utility of health service services and medication use in secondary chronic headache in the general population.

**Methods:**

An age and gender stratified cross-sectional epidemiological survey included 30,000 persons 30–44 years old. Diagnoses were interview-based. The International Classification of Headache Disorders 2^nd^ ed. was applied along with supplementary definitions for chronic rhinosinusitis and cervicogenic headache. Secondary chronic headache exclusively due to medication overuse was excluded.

**Results:**

One hundred and thirteen participants had secondary chronic headache. Thirty % had never consulted a physician, 70% had consulted their GP, 35% had consulted a neurologist and 5% had been hospitalised due to their secondary chronic headache. Co-occurrence of migraine or medication overuse increased the physician contact. Acute headache medication was taken by 84% and 11% used prophylactic medication. Complementary and alternative medicine was used by 73% with the higher frequency among those with than without physician contact.

**Conclusion:**

The pattern of health care utilisation indicates that there is room for improving management of secondary chronic headache.

## Background

The WHO initiated ‘Lifting the burden: the global campaign to reduce the burden of headache’ because headache is common, under-diagnosed and undertreated [1-3].

The International Classification of Headache Disorders 2^nd^ edition (ICHD-II) provides diagnostic criteria for headaches which are divided into primary and secondary forms [4].

The most common acute secondary headaches are induced by alcohol, fever, hunger and rhinosinusitis and are usually paroxysmal [5], but secondary chronic headache (≥ 15 days per month ≥ 3 months or ≥ 180 days/last year) is also common and medication overuse contributes to the problem [6,7].

Most headaches are self-managed [8], but headache is also one of the most common reasons for consulting a general practitioner (GP) and accounts for 4% of all GP consultations in the UK [9,10]. Approximately 20-30% of all new referrals to out-patients neurological departments are due to headache [9-12].

Headache has been suggested to be the most common new neurological symptom presented [13], and many neurological conditions include headache in the symptomatology, thus it also represents an important neurological differential diagnosis and may cause high use of health care services.

Epidemiological data on secondary headaches are largely lacking, and most information are from studies that have not had a main focus on secondary chronic headache. The International Headache Society‵s classification committee encourages further research in order to provide more knowledge and information of secondary headaches [4,14].

We have previously described the management of primary chronic headaches in the general population [15]. Our aim here was to investigate secondary chronic headache in the general population in order to evaluate utility of health services and medication use.

## Methods

**Figure 1:**
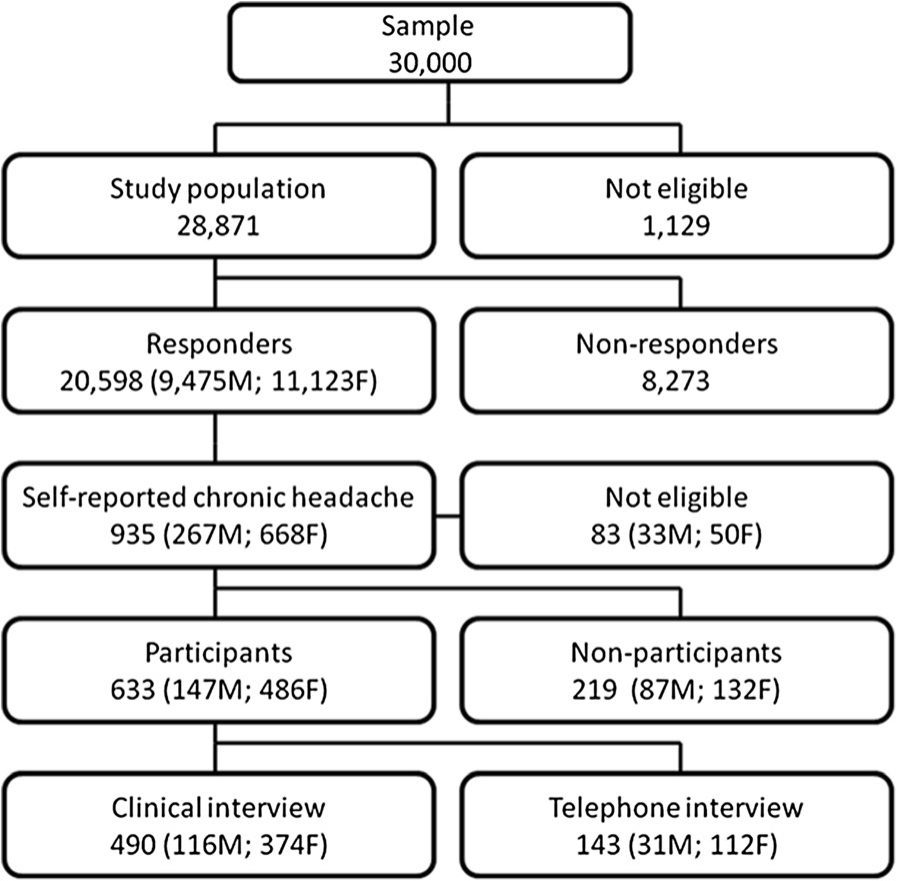
Flow chart of the participation.

Figure 1 shows a flow-chart of the study. The method has been described in more detail elsewhere [6,15].

### Sampling

A random age-stratified sample of 15 000 men and 15 000 women, 30–44 years old and residing in the 20 eastern municipalities in Akershus County, was drawn from the National Personal Registry by Statistics Norway. Akershus County has both rural and urban areas and is situated in close proximity to Oslo.

### Questionnaire

All persons in the sample received a mailed questionnaire with a standard letter containing information about the project. Apart from ensuring confidentiality and emphasizing the importance of participation, it was stated that the object was to study headache. The questions ‘How many days during the last month have you had headache?’ and ‘How many days during the last year have you had headache?’ were used to screen for chronic headache. If the questionnaire evoked no response, a second and subsequently a third reminder were issued.

### Clinical interview, physical and neurological examination

The study took place at the Akershus University Hospital in 2005. Persons with self-reported chronic headache who also consented by adding their telephone number on the questionnaire were invited to a clinical examination. Self-reported chronic headache was defined to be headache occurring ≥ 15 days within the last month and/or headache occurring ≥ 180 days within the last year. Inclusion required Norwegian languages skills. Two neurological residents (RBG and KA) experienced in headache diagnostics conducted all interviews and the physical and neurological examinations. Those unable to meet at the clinic were interviewed by telephone.

### Headache classification

All headaches were classified according to explicit diagnostic criteria of the ICHD-II and its relevant revisions [4,16-18]. Those with secondary chronic headache exclusively due to medication overuse were excluded.

We defined secondary chronic headache as secondary headache ≥15 days/month for at least 3 months, as the ICHD-II do not provide an explicit definition of frequency for secondary headaches. A more detailed description has been published elsewhere [6,19].

Chronic post-traumatic headache (CPTH) included head and whiplash traumas.

Cervicogenic headache (CEH) was classified according to the criteria of the Cervicogenic Headache International Study Group, requiring at least three criteria to be fulfilled not including blockade of the neck due to the non-interventional nature of our study (Table 1) [20].

**Table 1:** **Definition of cervicogenic headache **[20]

	
Major criteria	I. Symptoms and signs of neck involvement
	Ia. Precipitation of head pain, similar to the usually occurring one:
	Ia1) by neck movement and/or sustained, awkward head positioning, and/or:
	Ia2) by external pressure over the upper cervical or occipital region on the symptomatic side.
	Ib. Restriction of the range of motion (ROM) in the neck.
	Ic. Ipsilateral neck, shoulder or arm pain of a rather vague, non-radicular nature, or – occasionally – arm pain of a radicular nature.
	II. Confirmatory evidence by diagnostic anaesthetic blockades.
	III. Unilaterality of the head pain, without sideshift.
Head pain characteristics	IV. Moderate-severe, non-throbbing pain, usually starting in the neck. Episodes of varying duration, or: fluctuating, continuous pain.
Other characteristics of some importance	V. Only marginal effect or lack of effect of indomethacin. Only marginal effect or lack of effect of ergotamine and sumatriptan. Female sex. Not infrequent occurrence of head or indirect neck trauma by history, usually of more than only medium severity.
Other features of lesser importance	VI. Various attack-related phenomena, only occasionally present, and/or moderately expressed when present: a) nausea, b) phono- and photophobia, c) dizziness, d) ipsilateral “blurred vision”, e) difficulties swallowing, f) ipsilateral oedema, mostly in the periocular area.

It is obligatory that one or more of the phenomena Ia–Ic are present.

Headache attributed to chronic rhinosinusitis (HACRS) was defined according to the criteria established by the American Academy of Otolaryngology – Head and Neck Surgery (Table 2) [21] adding that the symptoms had persisted 12 weeks or more. Those with suspected HACRS were examined with anterior rhinoscopy and completed the Sino-Nasal questionnaire (SNOT-20) [22].

**Table 2:** **Definition of rhinosinusitis by the American Academy of Otolaryngology – Head and Neck Surgery **[21]

	
*Major factors*	Facial pain/pressure
	Nasal obstruction/blockage
	Nasal discharge/purulence/discolored postnasal drainage
	Hyposmia/anosmia
	Purulence in nasal cavity on examination
	Fever (acute rhinosinusitis)
*Minor factors*	Headache
	Fever (all nonacute)
	Halitosis
	Fatigue
	Dental pain
	Cough
	Ear pain/pressure/fullness

Two major factors or one major and two minor factors are required for the diagnosis. Of note, facial pain requires another major factor associated with it for diagnosis, as facial pain plus two minor factors is not deemed sufficient for diagnoses of rhinosinusitis.

Pregnancy related headache is not an ICHD-II diagnosis unless it is associated with eclampsia. We included 4 women who experienced headache exclusively during pregnancy. Two women had chronic headache > 3 months associated with pre-eclampsia, one had had headache during all 3 pregnancies and one had headache during her first pregnancy.

### Physician consultation

We defined four levels of contact due to headache, i.e. none (no physician contact), primary (GP), secondary (neurologist) and tertiary (hospitalisation). In Norway, a GP referral is a prerequisite for access to neurologists, while both GPs and neurologists can refer to the hospital.

### Complementary and alternative medicine (CAM) contacts

The CAM forms queried were acupuncture, chiropractic, homeopathy, naprapathy, physiotherapy, psychologist and psychomotor physiotherapy.

### Medication use and dependency

We asked about current medication use, and excluded medication used for other pain conditions. Medication overuse was defined according to the ICHD-II criteria for medication overuse headache (i.e. ≥ 15 days per month for simple analgesics and ≥ 10 days per months for triptans and ergotamines) [4,17].

To assess dependency-like behaviour in relation to headache medication, we used the SDS, which includes five questions designed to measure psychological dependence [23,24]. The questions apply to the headache medication taken within the last month. Each item is scored on a 4-point scale (0–3), and the total maximum score is 15. The method has been described in detail elsewhere [24].

### Data processing

Data from the interviews were directly entered using SPSS Data Entry 4.0 (SPSS Inc., Chicago, IL, USA) and statistical analyses were performed using SPSS 20.00 (SPSS Inc., Chicago, IL, USA). For descriptive data, proportions, means and confidence intervals (CI) are given. Pearson χ^2^ test was used for testing significance of group differences for categorical data, Fisher‵s exact test was used when appropriate. Student‵s T-test was used for numerical data. Significance levels were set at p < 0.05 and 95% CI were calculated. CI and probabilities are not given when n <5.

### Ethical issues

The Regional Committee for Medical Research Ethics and the Norwegian Social Science Data Services approved the study. All participants gave informed consent.

## Results

### Participants and headache diagnoses

The questionnaire response rate was 71% and the interview participation rate was 74%. Among the participants, 82% (93/113) had an interview and a physical and neurological examination at the clinic, whereas 18% (20/113) had an interview by telephone. Whether the participants had been interviewed in person or by phone made no difference to the frequency of the various headache diagnoses, medication use, medication overuse, physician or CAM contact.

A total of 113 participants (22% men and 78% women) had secondary chronic headaches not exclusively due to medication overuse.

Forty-two % had CPTH, 21% had CEH, 41% HACRS and 8% had other secondary chronic headache, i.e. 3 post-craniotomy, 1 diving related, 4 pregnancy related, and 1 post-meningitis.

The sum exceeds 100%, since the diagnoses are not mutually exclusive. Forty-one% had co-occurrence of migraine and 49% had co-occurrence of medication overuse.

### Physician consultation pattern

Table 3 shows the physician contact pattern.

**Table 3:** Contact and treatment pattern in relation to secondary headache diagnoses

	**CPTH without medication overuse (N = 25)**	**CPTH with medication overuse (N = 22)**	**All CPTH (N = 47)**	**CEH without medication overuse (N = 12)**	**CEH with medication overuse (N = 12)**	**All CEH (N = 24)**	**HACRS without medication overuse (N = 22)**	**HACRS with medication overuse (N = 24)**	**All HACRS (N = 46)**	**Other secondary chronic headache without medication overuse (N = 8)**	**Other secondary chronic headache with medication overuse (N = 1)**	**All Other secondary chronic headache (N = 9)**	**All secondary chronic headaches without medication overuse (N = 58)**	**All secondary chronic headaches with medication overuse (N = 55)**	**All secondary chronic headaches (N = 113)**
	**% (n)**	**% (n)**	**% (n)**	**% (n)**	**% (n)**	**% (n)**	**% (n)**	**% (n)**	**%( n)**	**% (n)**	**% (n)**	**% (n)**	**% (n)**	**% (n)**	**% (n)**
***Contact level***															
*None*	28 (7)	18 (4)	23 (11)	42 (5)	25 (3)	33 (8)	36 (8)	17 (4)	26 (12)	63 (5)	0 (0)	56 (5)	41 (24)	18 (10)	30 (34)
*Primary*	72 (18)	82 (18)	77 (36)	58 (7)	75 (9)	67 (16)	64 (14)	83 (20)	74 (34)	38 (3)	100 (1)	44 (4)	59 (34)	82 (45)	70 (79)
*Secondary*	40 (10)	59 (13)	49 (23)	8 (1)	50 (6)	29 (7)	27 (6)	29 (7)	28 (13)	0 (0)	100 (1)	11 (1)	28 (16)	44 (24)	35 (40)
*Tertiary*	8 (2)	5 (1)	6 (3)	0 (0)	8 (1)	4 (1)	5 (1)	4 (1)	4 (2)	0 (0)	100 (1)	11 (1)	5 (3)	5 (3)	5 (6)
***Complementary and alternative medicine***															
*Acupuncture*	52 (13)	55 (12)	53 (25)	58 (7)	33 (4)	46 (11)	36 (8)	38 (9)	37 (17)	13 (1)	0 (0)	11 (1)	41 (24)	42 (23)	42 (47)
*Chiropractic*	40 (10)	59 (13)	49 (23)	33 (4)	33 (4)	33 (8)	27 (6)	33 (8)	30 (14)	0 (0)	0 (0)	0 (0)	29 (17)	42 (23)	35 (40)
*Homeopathy*	12 (3)	23 (5)	17 (8)	25 (3)	8 (1)	17 (4)	14 (3)	17 (4)	15 (7)	0 (0)	0 (0)	0 (0)	12 (7)	16 (9)	14 (16)
*Naprapathy*	4 (1)	9 (2)	6 (3)	0 (0)	8 (1)	4 (1)	0 (0)	0 (0)	0 (0)	0 (0)	0 (0)	0 (0)	2 (1)	4 (2)	3 (3)
*Physiotherapy*	72 (18)	100 (22)	85 (40)	75 (9)	75 (9)	75 (18)	36 (8)	58 (14)	48 (22)	25 (2)	100 (1)	33 (3)	52 (30)	76 (42)	64 (72)
*Psychologist*	4 (1)	0 (0)	2 (1)	0 (0)	0 (0)	0 (0)	5 (1)	8 (2)	7 (3)	0 (0)	0 (0)	0 (0)	2 (1)	4 (2)	2 (2)
*Psychomotor physiotherapy*	4 (1)	5 (1)	4 (2)	0 (0)	8 (1)	4 (1)	0 (0)	4 (1)	2 (1)	0 (0)	0 (0)	0 (0)	2 (1)	5 (3)	4 (4)
*Any CAM*	76 (19)	100 (22)	87 (41)	75 (9)	83 (10)	79 (19)	64 (14)	67 (16)	65 (30)	25 (2)	100 (1)	33 (3)	64 (37)	82 (45)	73 (82)
***Medication use***															
*Acute medication*	68 (17)	100 (22)	83 (39)	75 (9)	100 (12)	88 (21)	82 (18)	100 (24)	91 (42)	50 (4)	100 (1)	56 (5)	69 (40)	100 (55)	84 (95)
*Prophylactic medication*	16 (4)	14 (3)	15 (7)	8 (1)	17 (2)	13 (3)	5 (1)	13 (3)	9 (4)	0 (0)	0 (0)	0 (0)	9 (5)	13 (7)	11 (12)

*CPTH* Chronic post-traumatic headache; *CEH* Cervicogenic headache; *HACRS* Headache attributed to chronic rhinosinusitis.

The diagnoses are not mutually exclusive, i.e. one person can have two or more headache diagnoses.

Thirty % (34/113) had never had contact with the health care system for their secondary chronic headache, while 70% (79/113) had had contact with their GP, 35% (40/113) had been referred to a neurologist and 5% (6/113) had been hospitalised for their secondary chronic headache. There was no gender difference in the physician contact pattern.

### Complementary and alternative medicine

Table 3 shows that 73% (82/113) had used CAM for their secondary chronic headache. Physiotherapy, acupuncture and chiropractic were most frequently used. Physiotherapy was most commonly used in CPTH, CEH and HACRS, i.e. 85%, 75% and 48%, respectively.

The use of CAM was significantly higher among those who had consulted a physician compared to those who had not (84% vs. 47%, p < 0.001).

### Co-occurrence of migraine

Co-occurrence of migraine increased health care utilisation (physician contact (p = 0.043), and hospitalisation (p = 0.04)).

The overall use of CAM was not significantly influenced by migraine, although there was a tendency for a higher usage of CAM among people with than without co-occurrence of migraine.

### Use of medication

Acute medication was used by 84%, and 19% used it on a daily basis. A higher proportion of participants with than without co-occurrence of migraine used acute medication (93% vs. 78%, p = 0.024), while there was no gender difference. People using acute medication had significantly more physician contact than people not using acute medication (89% vs. 74%, p = 0.045). People using acute medication had significantly more CAM use than people not using acute medication (89% vs. 71%, p = 0.019).

**Figure 2:**
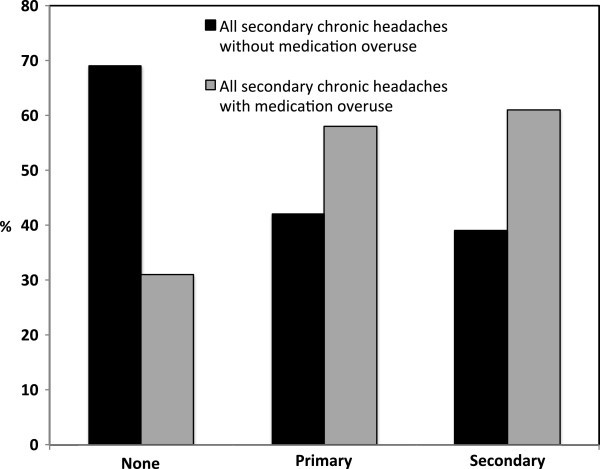
Physician contact levels for participants with secondary chronic headache without (dark grey) or with (light grey) medication overuse.

**Figure 3:**
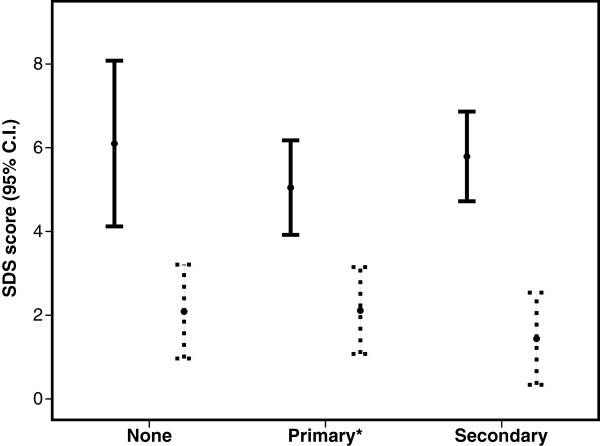
**Severity Dependence Scale (SDS) scores in participants with secondary chronic headache with (black) or without (dashed) medication overuse vs. contact level. **χ^2^, p < 0.0035 for all contact levels. *Primary contact level in this figure is defined as GP only (i.e. those participant with only GP contact without referral) due to illustrative purposes, to avoid overlap of groups and to allow adequate significant testing with χ2 test.

Contact with physicians was significantly influenced by medication overuse (82% vs. 59%, p = 0.007), and proportions with none, primary and secondary physician contact level was also different for those with compared to those without medication overuse (Figure 2). A higher proportion of those with than without medication overuse used CAM (82% vs. 64%, p = 0.032). The distribution of medication overuse was similar in different subtypes of secondary chronic headaches. Fifty-eight% overused simple analgesics, mainly paracetamol and/or ibuprofen and 31% overused combination analgesics, usually a combination of paracetamol and codeine. Of the latter 53% also overused simple analgesics. The physician contact level was not influenced by type of medication overuse. The SDS score was significantly higher in those with than without medication overuse for all levels of physician contact (Figure 3).

Prophylactic treatment was used by 11% (12/113) and was not influenced by gender or co-occurrence of migraine.

## Discussion

### Presentation of main findings

Two-thirds of those with secondary chronic headache consulted their GP for headache, and half of these had also consulted a neurologist. Approximately 40% had co-occurrence of migraine, and approximately 50% had medication overuse. The majority used acute medication, while prophylactic medication was rarely used. Medication use, medication overuse and high SDS score were associated with more physician contact, referral and use of CAM.

### Methodological considerations

The present study is based on recruitment from the general population. The large sample and high response rate should ensure representativity. The secondary chronic headaches CPTH, CEH and HACRS are frequent enough to ensure accurate descriptive statistics, while other types of secondary chronic headache are too infrequent for statistical analyses. The age range, though it may exclude some secondary headache types, was chosen explicitly to focus on a population without too much co-morbidity of non-headache disorders. Data from the Norwegian prescription registry indicate a high increase in drug prescriptions among people above 50 years [25]. This includes medication used for high blood pressure and pain killers for non-headache pain which both are is likely to influence the headache spectrum, a bias that we tried to avoid. Headache diagnoses are a challenge in people with chronic headache. To ensure precise diagnostic, two neurological residents experienced in headache diagnostics conducted all interviews. Complicated headache histories were discussed among the authors before classification. The different headache diagnoses were equally frequently by the two interviewers, suggesting that inter-observer variation was low. Whether the participants had been interviewed in person or by phone made no difference to the various outcomes. The ICHD-II classification of the secondary chronic headaches is a challenge since chronicity is not defined exhaustively [4]. Likewise, the diagnoses of CEH are not specific and the diagnosis HACRS is not part of the ICHD-II classification [20,21].

The data on medication use and health care utilisation are based on self-reports and therefore open to recall bias. Health registry data are, however, often incomplete and not necessarily more precise. Since there is no systematic registration of CAM, one has to rely on self-reports.

### Physician contact and use of complementary and alternative medicine

Surprisingly, even though high levels of specialist contact, high medication use, medication overuse and high proportions of CAM contacts seem to suggest a difficult headache situation for many of our participants, as many as 1/3 had no physician contact ever due to their headache. This figure is higher than the corresponding figure for primary chronic headaches where only 20% had no such contact [15]. This may be due to low expectations towards traditional medicine regarding secondary chronic headache, and/or on the contrary that secondary chronic headache is expected to be self-treatable. Another possible explanation might be that the secondary chronic headache is less disabling than migraine. Co-occurrence of migraine increased physician contact, and this might explain why some of those without co-occurrence of migraine rely on self-treatment for their secondary chronic headache.

The use of CAM was high in our population with secondary chronic headaches and the use of CAM was significantly higher among those who had consulted a physician compared to those who had not. We have no specific information on what the GPs and/or neurologists have done, but it is likely to assume that unsatisfactory pharmacological treatment lead to higher use of CAM. However, both pharmacological and CAM treatment followed the same pattern i.e. more medication use correlated with more CAM use. This suggests that some secondary chronic headache sufferers will try many treatment options to alleviate their headache.

The most frequent secondary chronic headaches (CPTH, CEH and HACRS) had surprisingly similar pattern of CAM use despite these are caused by different pathophysiological mechanisms. Physiotherapy was the most commonly used strategy in all these three groups, but to a much higher degree in people with CPTH/CEH probably due to the fact that these people have more neck and muscular pain and tenderness than people with HACRS. Usage of acupuncture, chiropractic and homeopathy was remarkably similar between the three groups. In relation to HACRS, this may suggest that this entity is rarely recognised by people or diagnosed by either physicians or by those practicing CAM. An alternative may be that rhinosinusitis problems are commonly present in other conditions with secondary headache, either by coincidence or by interactions based on medication use or other causal or interactive relationships such as sensitisation processes. We have previously described that HACRS may have a better prognosis than CPTH and especially CEH which support HACRS as a different entity [19,22]. The improvement among participants with HACRS was seen especially in those who had interventions for their rhinosinusitis and who had reduced their nasal decongestants and their headache medication overuse [22]. This would seem to support the presence of HACRS as a chronic headache entity distinct from CPTH and CEH and that a focus and referral practice reflecting this by traditional school medicine physicians may give a hope for a more effective treatment for a group of sufferers from secondary chronic headache.

### Use of medication

Use of acute headache medication is associated with physician contact and CAM treatment, but even at the “none” contact level more than 70% used acute headache medication. Altogether, almost 50% overused headache medication. In the most revised version of the headache classification this headache should be classified as *probable* MOH until detoxification has taken place, in which case it would be classified either as MOH (if improvement is seen) or as the remaining alternative, secondary chronic headache diagnosis (if no improvement is seen) [4,16-18]. However in a cross-sectional study such as the present one, both diagnoses must be given and a co-morbid situation considered. Medication overuse as well as SDS score related to the overuse was significantly associated with increasing contacts with health services, whether CAM or GPs/neurologists. Medication overuse seems to be either a marker of a complex chronic headache requiring increased support from the health services or causally associated with a worsening of the headache. The direction of causality of these associations cannot be determined in a cross-sectional study. The concept of medication overuse in secondary headaches is still debatable. Frequent use of medication, for instance, could be fully justified by a chronic inflammation or other conditions. In addition, the direction of a possibly causal association between medication overuse and the underlying headache becomes even more difficult to ascertain if the headache is assumed to be secondary to another, defined organic cause. However, simply the high co-occurrence of medication overuse seems to suggest the potential for trying detoxification also in the case of secondary chronic headaches. We have previously reported that reduced medication overuse is associated with improvement of CPTH and HACRS [19,22]. CEH did not improve after detoxification, but detoxification did not *worsen* the headache or have other negative effects [19].

In contrast to the high level of acute medication use stands the low level use of prophylactics. Which prophylactics that may be useful in secondary chronic headaches is not sufficiently described and should be addressed by further studies.

However, the 41% co-occurrence of migrainous headache may suggest a potential for using migraine prophylactics. It is suggested that migraineurs are undertreated and a substantial proportion of those who might benefit from migraine prevention do not receive it [26,27].

## Conclusion

Secondary chronic headache is a heterogeneous group of headache disorders with a high level of health care utilisation. The high level of neurologist contact, medication use/overuse and use of CAM indicate that this might be a complicated group to handle both in primary care and in neurologists setting. The treatment strategy should be focused on the type of secondary chronic headache. Detoxification of medication overuse is recommended and prophylactic treatment might also be effective, although the latter has not been documented in clinical trials.

## Competing interest

The authors declare that they have no competing interest.

## Authors’ contributions

MBR had the original idea for the study and planned the overall design. ESK prepared the initial draft and was the main author of the present manuscript. RBG and KA collected data. CL and MBR was involved in data analysis and interpretation and assisted in preparation of the manuscript. All authors have read, revised and approved the final manuscript.
